# Population Risk Factors for Severe Disease and Mortality in COVID-19 in the United States during the Pre-Vaccine Era: A Retrospective Cohort Study of National Inpatient Sample

**DOI:** 10.3390/medsci10040067

**Published:** 2022-12-04

**Authors:** Kavin Raj, Karthik Yeruva, Keerthana Jyotheeswara Pillai, Preetham Kumar, Ankit Agrawal, Sanya Chandna, Akhilesh Khuttan, Shalini Tripathi, Ramya Akella, Thulasi Ram Gudi, Abi Watts, Christian C Toquica Gahona, Umesh Bhagat, Surya Kiran Aedma, Ayesha Tamkinat Jalal, Shyam Ganti, Padmini Varadarajan, Ramdas G Pai

**Affiliations:** 1 Division of Cardiology, Department of Medicine, University of California Riverside School of Medicine, Riverside, CA 92521, USA; 2 Department of Internal Medicine, Merit Health River Region Hospital, Vicksburg, MS 39183, USA; 3 Department of Medicine, Kilpauk Medical College, Tamil Nadu 600010, India; 4 Department of Hospital Medicine, Cleveland Clinic Foundation, Cleveland, OH 44195, USA; 5 Department of Cardiac Hospital Medicine, University of Iowa, Iowa City, IA 52242, USA; 6 Department of Hospital Medicine, Carolina East Medical Center, New Bern, NC 28560, USA; 7 Department of Hospital Medicine, Pikeville Medical Center, Pikeville, KY 41501, USA; 8 Division of Cardiology, Department of Medicine, University of Texas Health Sciences Center at Houston, Houston, TX 77030, USA; 9 Division of Cardiology, Department of Medicine, Kansas University Medical Center, Kansas City, KS 66160, USA; 10 Department of Internal Medicine, Memorial Healthcare System, Hollywood, FL 33021, USA; 11 Department of Pulmonary Critical Care, Appalachian Regional Healthcare, Lexington, KY 40505, USA

**Keywords:** COVID-19 complications, COVID-19 mortality, socioeconomic disparities, COVID-19 risk factors, national inpatient sample (NIS), cardiovascular diseases

## Abstract

Background-Previous studies on coronavirus disease 2019 (COVID-19) were limited to specific geographical locations and small sample sizes. Therefore, we used the National Inpatient Sample (NIS) 2020 database to determine the risk factors for severe outcomes and mortality in COVID-19. Methods-We included adult patients with COVID-19. Univariate and multivariate logistic regression was performed to determine the predictors of severe outcomes and mortality in COVID-19. Results-1,608,980 (95% CI 1,570,803–1,647,156) hospitalizations with COVID-19 were included. Severe complications occurred in 78.3% of COVID-19 acute respiratory distress syndrome (ARDS) and 25% of COVID-19 pneumonia patients. The mortality rate for COVID-19 ARDS was 54% and for COVID-19 pneumonia was 16.6%. On multivariate analysis, age > 65 years, male sex, government insurance or no insurance, residence in low-income areas, non-white races, stroke, chronic kidney disease, heart failure, malnutrition, primary immunodeficiency, long-term steroid/immunomodulatory use, complicated diabetes mellitus, and liver disease were associated with COVID-19 related complications and mortality. Cardiac arrest, septic shock, and intubation had the highest odds of mortality. Conclusions-Socioeconomic disparities and medical comorbidities were significant determinants of mortality in the US in the pre-vaccine era. Therefore, aggressive vaccination of high-risk patients and healthcare policies to address socioeconomic disparities are necessary to reduce death rates in future pandemics.

## 1. Introduction

Coronavirus disease 2019 (COVID-19) is an ongoing pandemic caused by severe acute respiratory syndrome coronavirus 2 (SARS-CoV-2) [1]. There is a broad spectrum of disease manifestations, including asymptomatic shedding, acute upper respiratory tract infection, multilobar pneumonia, acute respiratory distress syndrome, and death [2]. The US was the most affected country in the pandemic, with 96.5 million cases and 1.06 million deaths [3].

Since the early pandemic, certain risk factors were well established with severe outcomes in COVID-19. This includes older age, certain demographic factors such as minority race, and specific comorbidities [4,5,6,7,8]. However, these studies were limited to specific geographical locations and small sample sizes. This limits the generalizability of these findings to the entire US population. NIS (National Inpatient Sample) is the largest national inpatient database in the US. Due to the large sample size and weighted design, the NIS database enables more accurate national estimates of the epidemiology of COVID-19 [9]. 

Therefore, we designed a retrospective cohort study with the newly launched NIS 2020 to study the epidemiology of COVID-19 in the pre-vaccine era in the US. Although we are past the pandemic’s peak, this data will help us understand the medical risk factors and social disparities in the pre-vaccine era. This knowledge will be crucial in the handling of the current pandemic and future pandemics.

## 2. Materials and Methods

### 2.1. Data Source 

The study was derived from the NIS 2020. The NIS is part of a family of databases and software tools developed for the Healthcare Cost and Utilization Project (HCUP). The NIS is the largest publicly available all-payer inpatient healthcare database designed to produce U.S. regional and national estimates of inpatient utilization, access, cost, quality, and outcomes. Unweighted, it contains data from around 7 million hospital stays each year. Weighted, it estimates around 35 million hospitalizations nationally. The NIS is drawn from all States participating in HCUP, covering more than 97 percent of the U.S. population. The NIS approximates a 20-percent stratified sample of discharges from U.S. community hospitals. The self-weighting design of the new version of NIS reduces the margin of error for estimates and delivers more stable and precise estimates than previous versions of the NIS.

### 2.2. Data Selection 

COVID-19 was classified by the Centre for Disease Control (CDC) into asymptomatic COVID-19, acute bronchitis and lower respiratory infections, pneumonia, and acute respiratory distress syndrome (ARDS) (Table S1/Table S2). Adult patients (age 18 years and above) with COVID-19 (ICD-10 code U07.1), except those who were transferred to another acute care hospital, were included. This was necessary as the outcome of interest (mortality and severe complications) may not have occurred in that hospitalization. 

### 2.3. Outcomes

Our primary outcome is determining the risk factors for severe disease and mortality related to COVID-19. Complicated COVID-19 is defined as any of the following- urinary ultrafiltration, acute liver failure, red blood cell (RBC) transfusion, non-invasive positive pressure ventilation including Bilevel Positive Airway Pressure (BIPAP), Continuous Positive Airway Pressure (CPAP) or High Flow Nasal Cannula (HFNC); mechanical ventilation, vasopressor use, septic shock, Extracorporeal Membrane Oxygenation (ECMO), or cardiac arrest. These variables were selected based on the variables used in the Sequential Organ Failure Assessment (SOFA) score. Secondary outcomes include studying the distribution of socioeconomic factors and comorbidities associated with different severities of COVID-19.

### 2.4. Definition of Variables 

NIS variables were used to identify patients’ demographic characteristics, including age, sex, insurance status, race, and income quartiles based on zip code and hospital characteristics (bed size, teaching status, and ownership). Certain variables such as complicated and uncomplicated hypertension, complicated and uncomplicated diabetes, solid cancer, chronic pulmonary disorders, liver disease, and rheumatoid arthritis/collagen vascular disorders were part of the exhauster comorbidity index and were used directly. The remaining variables were extracted using the International Classification of Diseases, Tenth Revision, and Clinical Modification (ICD-10-CM) diagnosis codes. Comorbidities were selected based on the CDC guidelines (Table S1/Table S2). 

### 2.5. Statistical Analysis 

Descriptive analysis was conducted to determine the mean for continuous variables and the proportion for categorical variables. As NIS is a survey dataset, the proportion and the total number of events are reported with confidence intervals. First, we studied the distribution of socioeconomic variables, comorbidities, and complications among different severities of COVID-19. Univariate logistic regression was performed to determine the predictors of severe complications and mortality associated with COVID-19. Variables with significant *p* values (*p* < 0.05) were used in the multivariate logistic regression to determine the independent predictors of mortality and complications in COVID-19. Analysis was carried out using StataCorp. 2021. Stata Statistical Software: Release 17. StataCorp LLC (College Station, TX, USA), BE version. Stata’s every command and appropriate weights were used in all estimations. The overall fit was assessed using Receiver Operative Curves (ROC), and sensitivity analysis was performed using the value package. The study was exempt from institutional review board approval as the database uses previously collected de-identified data.

## 3. Results

### 3.1. Distribution of Risk Factors among Varying Severities of COVID-19

A total of 1,678,995 (1,640,344–1,717,646) hospitalizations had COVID-19. After excluding pediatric patients (19,509, 95% CI 17,038–21,981) and transferred patients (50,505, 95% CI 47,981–53,029), a total of 1,608,980 (1,570,803–1,647,156) hospitalized patients were included. A total of 420,940 (410,052–431,827) patients had asymptomatic COVID-19, and 18,689 (17,511–19,868) patients had COVID-19 bronchitis and lowered respiratory tract infection (LRTI). A total of 1,173,040 (1,142,995–1,203,085) patients had COVID-19 pneumonia, and 94,804 (90,337–99,272) had COVID-19 ARDS. Overall, 222,490 (215,758–229,221) patients did not survive, which corresponds to a mortality rate of 13.8%. The mortality rate for COVID-19 pneumonia was 16.6%, and for COVID-19, ARDS was 54%. 

Compared with asymptomatic COVID-19 and COVID-19 bronchitis/LRTI, patients with COVID-19 pneumonia and COVID-19 ARDS were older and had fewer female patients. The COVID-19 ARDS group had a higher number of Hispanics compared with other groups. There were no significant differences in primary payer, zip code income quartiles, and hospital ownership. Large hospitals and urban teaching hospitals had more COVID-19 ARDS when compared with small and medium hospitals. Hospitals in the southern US had more COVID-19 cases in all subtypes (Table 1).

Stroke, chronic kidney disease (CKD), heart failure, cardiomyopathy, and ischemic heart disease were more prevalent in COVID-19 ARDS. Diabetes, dyslipidemia, malnutrition, OSA, overweight/obesity, long-term steroid/immunomodulator use, and hypertension showed an increasing prevalence with worsening severity. Substance abuse and nicotine use showed decreasing prevalence with worsening severity. COVID-19 LRTI and bronchitis were much more prevalent among patients with chronic pulmonary disorders. COVID-19 ARDS showed a higher proportion of severe liver disease when compared to other subtypes. The remaining variables did not show any clear trend with worsening severity (Table 2).

Exhausters comorbidity score showed an increasing trend both on a categorical and numerical scale with worsening disease severity. The mean length of stay (LOS) was 8.8 days for COVID-19 pneumonia and 19.7 days for COVID-19 ARDS. The mean hospitalization charges were 104,776 US dollars for COVID-19 pneumonia and 325,294 (US dollars for COVID-19 ARDS. As expected, all complications showed an increasing trend with worsening severity. Severe complications occurred in 78.3% of COVID-19 ARDS and 25% of COVID-19 pneumonia patients. The septic shock occurred in 8.8% of COVID-19 pneumonia and 42.4% of COVID-19 ARDS patients. Cardiac arrest occurred in 3.2% of COVID-19 pneumonia and 10.3% of COVID-19 ARDS patients (Table 3).

### 3.2. Risk Factors for Severe Complications in COVID-19 

On univariate analysis, ages 40–65 and age > 64 were associated with more complications when compared to the age group of 18 to 40 years. On multivariate analysis, the female sex was associated with lower odds of COVID-19 complications (aOR 0.72, 95% CI 0.70–0.73). Medicare (aOR 1.25, 95% CI 1.21–1.29) and Medicaid (aOR 1.10, 95% CI 1.06–1.14) were associated with higher complications when compared with private insurance. When compared with whites, all races were associated with higher odds of COVID-19 complications. The native American race had the highest risk for complications (aOR 1.81, 95% CI 1.63–2.02). Geographical locations with higher income (>25th percentile) were associated with lower odds of COVID-19 complications when compared with low income (<25th percentile). Medium and large hospitals were associated with higher odds of COVID-19 complications when compared with small hospitals. Similarly, urban non-teaching and urban teaching hospitals were associated with higher odds of COVID-19 complications when compared with rural hospitals. Private-not-profit hospitals were associated with lower odds for COVID-19 complications when compared with government hospitals (Table 4).

Stroke, malnutrition, and liver disease had an adjusted odds ratio (aOR) of more than 2. CKD, heart failure, primary immunodeficiency, long-term steroid/immunomodulatory use, COPD, complicated DM, obstructive sleep apnea, and elixhauser comorbidity index had an aOR of more than 1.24 for COVID-19 complications. Cardiomyopathy, uncomplicated DM, complicated hypertension, solid cancer, overweight and obesity, other chronic pulmonary disorders, and rheumatoid arthritis/collagen vascular disorders had an aOR from 1.01 to 1.24 (Table 5).

### 3.3. Risk Factors for Mortality in COVID-19 

Elderly patients (age 65 and above) had the highest odds for mortality, followed by the age group 40 to 64 years. The female sex was associated with lower mortality (aOR 0.69, 95% CI 0.68–0.71). Medicaid and self-pay were associated with slightly higher odds of mortality when compared with private insurance. When compared with whites, blacks had similar mortality (aOR 1.03, 95% CI 0.99–1.07). Hispanics, Asian/pacific islanders, and other races had higher odds of mortality. Native Americans had the higher odds of mortality among all races (aOR 1.98, 95% CI 1.73–2.27). Geographical locations with higher income (>25th percentile) were associated with lower odds of mortality when compared with low income (<25th percentile). 

Medium and large hospitals were associated with higher mortality when compared with small hospitals. Similarly, urban non-teaching and urban teaching hospitals were associated with lower mortality when compared with rural hospitals. Hospitals in all geographical locations had lower mortality when compared with hospitals in the northeast region. Private-owned hospitals were associated with lower mortality when compared with government hospitals (Table 6).

On multivariate analysis, stroke and liver disease had an aOR of more than 2 for mortality. Cerebral palsy, heart failure, complicated DM, malnutrition, primary immunodeficiency, long-term steroid/immunomodulatory use, COPD, and an aOR of more than 1.24 for mortality. CKD, cardiomyopathy, uncomplicated DM, solid cancer, overweight and obesity, rheumatoid/collagen vascular disorders, and other chronic pulmonary disorders had an aOR of 1.01 to 1.24 for mortality. Cardiac arrest, septic shock, and intubation had the highest odds for mortality (Table 7).

## 4. Discussion

Our study is one of the largest studies on the epidemiology of COVID-19. While it has strengthened some of the results from the prior smaller studies, it has also shown some key differences. In our study of 1.6 million COVID-19 hospitalizations, we stratified the mortality depending on the COVID-19 severity, which is unique. Interestingly, we noticed a 16.6% mortality rate in COVID-19 pneumonia patients and a heightened mortality rate of 54% in COVID-19-associated ARDS patients. This is consistent with prior literature, which showed an in-hospital mortality rate of 11.4% in the US during the pre-vaccine era [10]. Consistent with the prior literature, age, male sex [11], non-Caucasian race, and certain comorbidities [12] were associated with higher odds of experiencing complicated COVID-19 and death.

Our study showed that the elderly population (age above 65) has higher complications and mortality when compared to middle-aged adults. Extremes of age are known to have a weaker immune system predisposing to COVID-19 complications [13]. Anyway, elderly patients have more comorbidities that predispose them to COVID-19 complications. Similar to the multiple prior works of literature [14,15], the male sex is associated with increased complications and mortality associated with COVID-19 despite adjusting for other risk factors. Although several mechanisms have been proposed, estrogen is shown to suppress pro-inflammatory cytokines such as IL-6, which is implicated in pulmonary vessel leakage and lung damage seen with COVID-19 [16].

Some studies have shown minority races to have a worse prognosis [17,18], whereas other studies have shown no such association [19,20]. Although these studies may be limited to their respective geographical locations, our study is much more generalizable and shows that all non-white races have higher odds of COVID-19 complications. However, when compared with whites, blacks had similar mortality. Hispanics, Asian/pacific islanders, native Americans, and other races had higher mortality when compared with whites. Native Americans had the highest complications and COVID-19-related mortality, consistent with the current literature [21].

Although the exact cause is unclear, several reasons have been proposed, such as high poverty rates, high prevalence of risk factors, and lack of access to healthcare among minority races. However, our analysis showed that despite adjustment for these factors, there were higher odds for complications and mortality. This suggests other factors, such as genetic predisposition, may be seen in non-white races.

Larger and urban hospitals likely have higher mortality because they were referral centers catering to the sickest patients. It is unclear why hospitals in the northeast had higher complications and mortality. One possible explanation was that the northeastern US is one of the most densely populated areas and was the pandemic’s epicenter, which may have led to resource overutilization [22]. The association between low-income zip code locations, government insurance or no insurance, and government hospitals with increased morbidity and mortality points to the deficiency in the US healthcare system to effectively care for the economically underprivileged during the pandemic [23].

Similar to the prior literature, stroke, CKD, heart failure, malnutrition, primary immunodeficiency, long-term steroid/immunomodulatory use, complicated DM, and liver disease were strong predictors of mortality [11,24,25]. Anyway, uncomplicated DM, cardiomyopathy, solid cancer, COPD, and other chronic pulmonary disorders were also predictors of COVID-19 mortality in our study. The poor cardiopulmonary health and impaired immune function associated with these diseases are likely responsible for this increased risk [26].

Diabetes is a well-established risk factor for severe COVID-19 [27,28]. Our study is consistent with a prior study which shows that complicated diabetes mellitus is a more decisive risk factor for COVID-19 mortality than uncomplicated diabetes mellitus [11]. Insulin resistance enhances pro-inflammatory cytokine production and a worse prognosis [28]. Hypertension is the most common comorbidity and is not associated with increased mortality risk, as seen in our study and prior studies [11]. With regards to stroke, COVID-19 can increase the risk of stroke, and stroke can increase the risk of severe COVID-19, as seen in our study [29].

Chronic liver disease was the most significant risk factor for COVID-19 severity. A meta-analysis by CDC showed a similar pooled odds ratio for mortality [30]. Immune dysregulation, coagulopathy, and intestinal dysbiosis have been postulated as potential reasons. Cancer is associated with worse adverse outcomes in patients with COVID-19 [31]. The pathophysiology stems from the immunosuppressive state as a result of malignancy and anti-cancer treatment modalities putting such a cohort at higher risk for adverse prognosis in COVID-19 infection [32,33]

Our study shows that being overweight and obese is weakly associated with increased COVID-19 mortality (aOR 1.07, 95% CI 1.03–1.11). Prior literature showed that obesity is a strong risk factor for COVID-19 mortality, and the risk increases with increasing BMI [11,24]. One reason for the weaker association in our study could be the adjustment for complications of obesity, such as diabetes and chronic pulmonary disorders, which were significant for mortality. This may be the reason for the lack of association of smoking and hypertension for mortality as we adjusted for cardiovascular diseases such as heart failure and ischemic heart disease. This was shown in this study [11]. Initially, hypertension showed a negative association with mortality. However, hypertension was significantly associated with mortality after removing obesity and diabetes from the multivariable model. The lack of association of transplant status with mortality could also be due to the adjustment for immunosuppressants/steroid use.

The prior literature has shown intellectual disability, paralysis, mood or psychotic disorders, asthma, HIV/AIDS, and cystic fibrosis to be associated with increased mortality [26]. However, our study failed to demonstrate this association. Cerebral palsy was the only chronic neurological disease associated with increased mortality in our study. Although OSA was associated with higher complications, it was not associated with higher mortality. Consistent with the literature, the number of comorbidities predicted severe outcomes, as shown by the strong association of the elixhauser comorbidity index on a categorical and numerical scale. Cardiac arrest, septic shock, and intubation had very high odds of mortality. Therefore, it is important to aggressively manage these patients and consider hospice in case of poor functional outcomes as appropriate.

## 5. Limitations

Our study has some limitations. First, NIS is an inpatient database and does not track patients post-discharge. This is not captured if a patient was discharged alive but died at home or at a rehab facility. Second, NIS, being an administrative database, relies on ICD-10 codes, which is inferior to manual chart review. There is a lack of information on imaging studies, laboratory investigations, treatment regimens, and vaccination status, which could affect outcomes. As with any observational study, association does not mean causation, and conclusions should be drawn cautiously. Nevertheless, NIS is an extensive database that can reveal socioeconomic and medical factors in healthcare utilization and patient outcomes.

## 6. Conclusions

COVID-19 is associated with high inpatient mortality in the US during the pre-vaccination era. Racial and Socioeconomic predictors of health appear to play a crucial role in this pandemic. It is imperative that we develop healthcare policies to address these gaps to alter the course of future pandemics. The number of medical comorbidities and certain comorbidities such as stroke, complicated DM, CKD, Chronic liver disease, and malnutrition appear to be associated with the highest risk for COVID-19 complications and mortality. These patients need aggressive preventive care such as vaccination and close monitoring to prevent complications.

## Figures and Tables

**Table 1:** The proportion of Socioeconomic Predictors for COVID-19 Severity.

	Asymptomatic COVID-19	COVID-19 Bronchitis and LRTI	COVID-19 Pneumonia	COVID-19 ARDS
Age (mean)	59.9 (59.6–60.3)	61.8 (61.2–62.5)	64.4 (64.2–64.6)	64.1 (63.8–64.3)
Age categories				
18–40	22.8 (22.1–23.4)	15.6 (14.4–17)	8.7 (8.5–8.9)	6.3 (5.9–6.7)
41–64	29 (28.6–29.4)	35.3 (33.7–37)	38 (37.7–38.4)	40.9 (40.1–41.7)
65 and above	46.5 (45.8–47.2)	46.9 (45.1–48.7)	51.1 (50.7–51.5)	50.1 (49.3–51)
Female sex	55.3 (54.8–55.7)	53.2 (51.4–54.9)	45.5 (45.2–45.8)	38.9 (38.1–39.6)
Primary payer				
Medicare	48.8 (48.1–49.6)	48.9 (47–50.8)	51.4 (50.9–52)	49.8 (48.8–50.8)
Medicaid	20.1 (19.4–20.8)	16.7 (15.1–18.4)	12.3 (11.9–12.8)	15 (14.2–15.8)
Private insurance	22.5 (21.9–23)	26.8 (25.1–28.5)	27.6 (27.1–28.2)	27.1 (26.1–28)
Self-pay	4.2 (3.9–4.5)	3.4 (2.7–4.2)	3.6 (3.3–3.9)	3.3 (2.9–3.8)
No charge	0.3 (0.2–0.3)	0.1 (0.05–0.3)	0.2 (0.1–0.3)	0.1 (0.1–0.2)
Other	3.9 (3.6–4.2)	3.8 (3.2–4.6)	4.5 (4.3–4.8)	4.5 (4.1–4.9)
Race				
Whites	53.8 (52.5–55)	50.1 (47.5–52.7)	49.7 (48.6–50.9)	43.3 (41.6–44.9)
Blacks	20.3 (19.3–21.2)	19.6 (18–21.2)	18.6 (17.8–19.4)	18.2 (17–19.4)
Hispanics	18.8 (17.8–19.8)	22.7 (20.3–25.2)	22.5 (21.4–23.5)	26.8 (25.2–28.4)
Asian or pacific islander	2.4 (2.2–2.6)	2.5 (2–3.1)	3.5 (3.2–3.7)	4.3 (3.9–4.8)
Native American	0.8 (0.7–0.9)	0.6 (0.4–1)	1 (0.8–1.2)	2 (1.5–2.6)
Others	3.7 (3.4–4.1)	4.3 (3.4–5.3)	4.4 (4–4.8)	5.2 (4.5–5.9)
Zip Income Quartile				
0–25th percentile	34.6 (33.4–35.8)	34.1 (32–36.3)	33.7 (32.6–34.9)	34.2 (32.5–35.8)
26th to 50th percentile	27.4 (26.6–28.3)	28.7 (26.9–30.5)	26.9 (26.1–27.7)	26.9 (25.8–28.1)
51st to 75th percentile	21.8 (21.1–22.5)	20.6 (19.1–22.2)	22.4 (21.6–23.1)	22.4 (21.3–23.4)
76th to 100th percentile	16 (15.1–16.9)	16.4 (14.8–18.2)	16.8 (15.9–17.8)	16.3 (15.1–17.7)
Hospital bed size				
Small	23.2 (22.2–24.2)	23.9 (21.5–26.5)	24 (22.9–25.1)	19.3 (17.7–21.1)
Medium	27.8 (26.7–28.9)	30.4 (27.6–33.4)	29.4 (28.3–30.5)	27.4 (25.3–29.5)
Large	48.9 (47.6–50.2)	45.5 (42.3–48.7)	46.4 (45.2–47.7)	53.2 (50.8–55.5)
Hospital type				
Rural	10 (9.5–10.6)	10.5 (9–12.2)	8.9 (8.4–9.4)	5.2 (4.5–6)
Urban non-teaching	16.2 (15.4–17)	21.6 (19.2–24.2)	19.4 (18.5–20.3)	14.1 (12.8–15.6)
Urban teaching	73.6 (72.6–74.6)	67.8 (64.9–70.5)	71.5 (70.5–72.8)	80.6 (79–82.1)
Hospital region				
Northeast	19.7 (18.6–20.9)	16.9 (14.2–20)	17.8 (16.8–18.9)	21 (19.-23.2)
Midwest	23.7 (22.7–24.8)	20.9 (18.9–23.2)	21.5 (20.6–22.5)	23.1 (21.2–25.1)
South	40.3 (39.1–41.6)	45.6 (42.5–48.8)	41.4 (40.1–42.6)	34.4 (32.3–36.6)
West	16 (15.2–16.9)	16.3 (14.3–18.5)	19.1 (18.1–20.1)	21.3 (19.5–23.3)
Hospital control				
Government, non-federal	13.6 (12.7–14.5)	12.4 (9.9–15.4)	11.4 (10.7–12.1)	12 (10.7–13.4)
Private, not-profit	74 (72.9–75)	67.8 (64.5–71)	73 (72–74.1)	78.3 (76.4–80)
Private, invest-own	12.3 (11.6–13)	19.6 (17.2–22.4)	15.4 (14.6–16.3)	9.6 (8.4–11)

**Table 2:** The proportion of Comorbidities in COVID-19.

	Asymptomatic COVID-19 Percentage (95% CI)	COVID-19 Bronchitis and LRTI Percentage (95% CI)	COVID-19 Pneumonia Percentage (95% CI)	COVID-19 ARDS Percentage (95% CI)
Stroke	1.8 (1.7–1.9)	0.7 (0.5–1.1)	1.1 (1–1.1)	2.9 (2.7–3.2)
CKD	20.1 (19.7–20.5)	18 (16.7–19.3)	20.9 (20.6–21.2)	22.7 (22–23.5)
All Disability *	2.1 (2–2.2)	1.6 (1.2–2.1)	1.1 (1.1–1.2)	1.5 (1.3–1.7)
Heart failure, cardiomyopathy and ischemic heart disease	3 (2.9–3.1)	2.4 (1.9–2.9)	2.8 (2.7–2.8)	3.5 (3.2–3.8)
Mood or psychotic disorders	1.2 (1.1–1.3)	0.6 (0.4–1)	0.6 (0.6–0.6)	0.6 (0.5–0.7)
HIV/AIDS	0.7 (0.6–0.8)	0.6 (0.4–0.9)	0.5 (0.5–0.6)	0.5 (0.4–0.6)
Diabetes	33.3 (32.9–33.8)	36.6 (35.1–38.2)	42.1 (41.8–42.5)	47.7 (46.8–48.5)
Hypertension	58.8 (58.1–59.4)	62.7 (60.9–64.4)	67.1 (66.7–67.5)	66.7 (65.7–67.6)
Solid cancer	3.7 (3.5–3.9)	2.7 (2.2–3.3)	2.4 (2.3–2.5)	2.1 (1.9–2.3)
Dyslipidemia	35.2 (34.7–35.8)	38.2 (36.6–39.9)	41.6 (41.2–42.1)	40.9 (39.9–41.9)
Overweight and obesity	17.8 (17.4–18.2)	25.1 (23.6–26.6)	28.5 (28–29)	35.3 (34.3–36.2)
Malnutrition	14.8 (14.4–15.1)	14.2 (12.8–15.6)	15.9 (15.5–16.2)	29.9 (28.8–30.9)
Primary immunodeficiency	0.1 (0.08–0.1)	0.1 (0.08–0.3)	0.1 (0.1–0.1)	0.2 (0.1–0.3)
Nicotine abuse	9.4 (9.1–9.6)	8.5 (7.7–9.5)	5.3 (5.2–5.5)	4.2 (3.9–4.5)
Sickle cell and thalassemia	0.1 (0.1–0.1)	0.08 (0.02–0.2)	0.1 (0.09–0.1)	0.07 (0.04–0.1)
Any transplant	0.7 (0.7–0.8)	0.5 (0.3–0.7)	0.7 (0.7–0.8)	0.9 (0.8–1.1)
Substance abuse	4.3 (4.1–4.5)	2.7 (2.2–3.3)	1.6 (1.6–1.7)	2 (1.8–2.2)
Tuberculosis	0.03 (0.02–0.04)	0.02 (0.003–0.1)	0.01 (0.01–0.02)	0.02 (0.01–0.06)
Long term steroid or immunomodulators	8.9 (8.7–9.2)	13.8 (12.7–15)	14.6 (14.4–14.9)	19.3 (18.7–20)
Chronic pulmonary disorders	18.9 (18.5–19.3)	47.8 (45.4–50.2)	22.9 (22.6–23.2)	22.6 (21.9–23.4)
Liver disease	5.5 (5.3–5.7)	4.8 (4.1–5.6)	5.4 (5.3–5.5)	10.7 (10.2–11.2)
Rheumatoid arthritis or collagen vascular disease	2.6 (2.5–2.7)	3.2 (2.6–3.8)	3 (2.9–3.1)	3.1 (2.9–3.4)
Obstructive sleep apnea	6 (5.8–6.3)	8.5 (7.6–9.5)	8.8 (8.6–9.1)	10.6 (10–11.1)
Mean Elixhauser comorbidity index	3.4 (3.3–3.4)	3.7 (3.6–3.8)	3.8 (3.7–3.8)	4.8 (4.7–4.8)
Elixhauser comorbidity index categories				
≤3	53.8 (53.2–54.4)	48.1 (46.1–50.1)	47.8 (47.3–48.2)	27 (26.1–28)
4–6	35.8 (35.3–36.3)	40.2 (38.4–42)	40.1 (39.8–40.4)	51.6 (50.8–52.5)
>6	10.3 (10–10.6)	11.6 (10.5–12.8)	12 (11.7–12.3)	21.2 (20.3–22.1)
Mean length of stay	5.8 (5.7–5.9)	7.6 (7.2–7.9)	8.8 (8.7–8.9)	19.7 (19.3–20.1)
Mean total charge	55,813 (54,288–57,339)	80,833 (73,720–87,947)	104,776 (101,927–107,625)	325,294 (309,542–341,047)

* All disabilities include Severe IQ disability, iodine deficiency, Downs Syndrome and Autism, ADHD, Cerebral Palsy, and Paralysis.

**Table 3:** The proportion of Complications in COVID-19.

	Asymptomatic COVID-19	COVID-19 Bronchitis and LRTI	COVID-19 Pneumonia	COVID-19 ARDS
Severe sepsis	2.3 (2.2–2.5)	3.5 (2.9–4.2)	5.5 (5.2–5.8)	8.2 (7.6–8.8)
Septic shock	2.6 (2.5–2.7)	4.3 (3.7–5.1)	8.8 (8.6–9)	42.4 (41.2–43.6)
Acute kidney injury	22.7 (22.3–23.1)	21.9 (20.5–23.5)	30.7 (30.4–31.1)	60.2 (59.2–61.1)
Urinary filtration	3.8 (3.6–4)	3.1 (2.6–3.8)	5.5 (5.4–5.7)	17.1 (16.3–17.9)
RBC transfusion	3.7 (3.5–3.9)	2.9 (2.4–3.5)	3.5 (3.3–3.7)	10.7 (9.8–11.7)
Vasopressor need	0.9 (0.8–1)	1.4 (1–1.9)	3.1 (2.9–3.4)	18 (16.3–19.8)
Acute respiratory failure	22.4 (21.8–23)	40.7 (38.3–43.1)	64.8 (64.2–65.4)	
BIPAP/CPAP and HFNC ^	1.7 (1.6–1.8)	4.1 (3.4–4.8)	7.7 (7.4–8.1)	18.1 (17–19.4)
Mechanical Ventilation	3.2 (3–3.3)	6.6 (5.8–7.6)	12.6 (12.4–12.9)	59.5 (58.2–60.9)
Acute liver failure	1 (0.9–1.1)	0.6 (0.4–0.9)	1.5 (1.4–1.5)	6.5 (6.1–6.9)
Extracorporeal membrane oxygenation	0.02 (0.01–0.04)	0.2 (0.1–0.5)	0.2 (0.2–0.2)	2.3 (1.9–2.8)
Cardiac arrest	1.1 (1.1–1.2)	1.5 (1.1–2)	3.2 (3–3.3)	10.3 (9.7–10.8)
Composite complications *	13.1 (12.7–13.4)	14.7 (13.5–16)	25 (24.7–25.4)	78.3 (77.3–79.3)

* urinary filtration, Acute Liver Failure, RBC transfusion, BIPAP/CPAP or HFNC, Mechanical Ventilation, Vasopressor, Septic Shock, ECMO and Cardiac Arrest, ^ BIPAP: Bilevel Positive Airway Pressure, CPA: Continuous Positive Airway Pressure, HFNC: High Flow Nasal Cannula.

**Table 4:** Socioeconomic Predictors of Complicated COVID-19.

	Uncomplicated COVID-19 1,255,350 (1,225,394–1,285,306)	Complicated COVID-19 353,629 (343,525–363,734)	Unadjusted		Adjusted *	
	Proportion (95% CI)	Proportion (95% CI)	OR (95% CI)	*p*-Value	OR (95% CI)	*p*-Value
Age	62.4 (62.2–62.6)	65.9 (65.8–66.1)	1.01 (1.01–1.01)	0.00	1.00 (1.00–1.00)	0.00
Age categories (years)						
18–40	14.2 (13.9–14.5)	6.2 (6–6.5)	Reference			
41–64	35.7 (35.4–36)	35.5 (35–35.9)	2.25 (2.17–2.33)	0.00		
65 and above	48.2 (47.7–48.7)	55.8 (55.2–56.3)	2.61 (2.52–2.71)	0.00		
Female sex	49.9 (49.7–50.2)	41.7 (41.3–42.1)	0.71 (0.70–0.73)		0.72 (0.70–0.73)	0.00
Insurance status						
Private insurance	48.3 (47.8–48.9)	59.2 (58.5–59.9)	Reference		Reference	
Medicare	14.7 (14.2–15.2)	13.6 (13–14.1)	1.67 (1.63–1.72)	0.00	1.25 (1.21–1.29)	0.00
Medicaid	27.9 (27.4–28.4)	20.4 (19.8–21)	1.26 (1.22–1.31)	0.00	1.10 (1.06–1.14)	0.00
Self-pay	4 (3.7–4.3)	2.8 (2.5–3)	0.95 (0.89–1.01)	0.10	0.90 (0.85–0.96)	0.02
No charge	0.2 (0.2–0.4)	0.1 (0.1–0.2)	0.92 (0.71–1.19)	0.54	0.83 (0.65–1.07)	0.15
Other	4.6 (4.3–4.8)	3.6 (3.3–3.9)	1.08 (1.02–1.14)	0.00	0.96 (0.90–1.02)	0.19
Race						
Whites	52.2 (51.3–53.3)	45.7 (44.4–47)	Reference		Reference	
Blacks	18.2 (17.5–19)	22 (21–23)	1.37 (1.33–1.42)	0.00	1.24 (1.19–1.28)	0.00
Hispanics	21.2 (20.2–22.2)	22.5 (21.4–23.7)	1.21 (1.17–1.25)	0.00	1.34 (1.29–1.39)	0.00
Asian or pacific islander	3.1 (2.9–3.3)	3.6 (3.3–3.9)	1.33 (1.26–1.40)	0.00	1.44 (1.36–1.52)	0.00
Native American	0.9 (0.7–1)	1.3 (1–1.6)	1.65 (1.48–1.85)	0.00	1.81 (1.63–2.02)	0.00
Others	4.1 (3.8–4.5)	4.6 (4.2–5.1)	1.26 (1.19–1.34)	0.00	1.37 (1.30–1.45)	0.00
Zip Income quartile						
0–25th percentile	33.2 (32.1–34.3)	36.9 (35.5–38.2)	Reference		Reference	
26th to 50th percentile	27.2 (26.5–28)	26.5 (25.6–27.3)	0.87 (0.84–0.90)	0.00	0.92 (0.89–0.95)	0.00
51st to 75th percentile	22.5 (21.8–23.2)	21.3 (20.5–22.1)	0.85 (0.82–0.88)	0.00	0.87 (0.84–0.90)	0.00
76th to 100th percentile	16.9 (16–17.9)	15.2 (14.3–16.2)	0.80 (0.77–0.84)	0.00	0.84 (0.80–0.87)	0.00
Hospital bed size						
Small	24.5 (23.5–25.6)	21 (19.8–22.2)	Reference		Reference	
Medium	28.9 (27.9–29.9)	29.5 (28.2–30.7)	1.19 (1.13–1.25)	0.00	1.16 (1.10–1.22)	0.00
Large	46.5 (45.3–47.7)	49.4 (48–50.8)	1.24 (1.18–1.30)	0.00	1.24 (1.18–1.31)	0.00
Hospital type						
Rural	10 (9.5–10.5)	6.6 (6–7.2)	Reference		Reference	
Urban non-teaching	18.9 (18–19.7)	17.4 (16.5–18.4)	1.39 (1.29–1.50)	0.00	1.34 (1.24–1.45)	0.00
Urban teaching	71 (70.1–72)	75.8 (74.7–76.9)	1.61 (1.50–1.72)	0.00	1.52 (1.41–1.64)	0.00
Hospital region						
Northeast	17.8 (16.8–18.8)	20.4 (19.1–21.8)	Reference		Reference	
Midwest	22.6 (21.7–23.6)	20.5 (19.3–21.6)	0.78 (0.74–0.84)	0.00	0.72 (0.67–0.77)	0.00
South	41.4 (40.2–42.6)	39.9 (38.6–41.3)	0.84 (0.79–0.88)	0.00	0.80 (0.75–0.85)	0.00
West	18 (17.1–19)	19 (17.9–20.1)	0.91 (0.86–0.96)	0.00	0.81 (0.76–0.87)	0.00
Hospital control						
Government, non-federal	11.9 (11.2–12.6)	12.2 (11.4–13.2)	Reference		Reference	
Private, not-profit	73.7 (72.7–74.6)	72 (70.7–73.2)	0.95 (0.89–1.00)	0.07	0.87 (0.82–0.93)	0.00
Private, invest-own	14.3 (13.6–15.1)	15.6 (14.7–16.6)	1.06 (0.99–1.13)	0.06	1.00 (0.93–1.07)	0.96

* Unweighted ROC for multivariate model = 0.72.

**Table 5:** Comorbidities which Predict Complicated COVID-19.

	Uncomplicated COVID-19	Complicated COVID-19	Unadjusted		Adjusted ^	
	Proportion (95% CI)	Proportion (95% CI)	OR (95% CI)	*p*-Value	OR (95% CI)	*p*-Value
Stroke	0.9 (0.9–1)	2.5 (2.4–2.7)	2.73 (2.56–2.91)	0.00	2.48 (2.28–2.69)	0.00
Chronic kidney disease	16.3 (16–16.5)	36.2 (35.7–36.8)	2.91 (2.84–2.98)	0.00	1.80 (1.74–1.87)	0.00
Severe IQ disability, iodine def, downs syndrome and autism	0.1 (0.1–0.1)	0.1 (0.1–0.1)	0.84 (0.66–1.07)	0.23	1.02 (0.78–1.33)	0.85
ADHD *	0.3 (0.3–0.4)	0.1 (0.1–0.2)	0.44 (0.36–0.53)	0.00	0.72 (0.58–0.88)	0.00
Cerebral palsy	0.3 (0.3–0.3)	0.3 (0.2–0.3)	1.00 (0.87–1.15)	0.96	1.33 (1.13–1.56)	0.00
Paralysis	0.5 (0.5–0.6)	1 (0.9–1.1)	1.90 (1.74–2.08)	0.00	1.01 (0.89–1.13)	0.88
Heart failure	13.4 (13.2–13.6)	26.8 (26.3–27.2)	2.35 (2.30–2.41)	0.00	1.42 (1.38–1.47)	0.00
Cardiomyopathy	2.3 (2.2–2.4)	4.7 (4.6–4.9)	2.10 (2.01–2.20)	0.00	1.20 (1.14–1.26)	0.00
Ischemic heart disease	16.6 (16.4–16.9)	22.5 (22.1–22.9)	1.45 (1.41–1.48)	0.00	0.92 (0.90–0.95)	0.00
Mood or psychotic disorders	0.8 (0.7–0.8)	0.7 (0.6–0.8)	0.85 (0.77–0.94)	0.00	0.90 (0.81–1.01)	0.09
HIV/AIDS	0.5 (0.5–0.6)	0.7 (0.6–0.8)	1.24 (1.12–1.38)	0.00	0.92 (0.81–1.04)	0.21
Uncomplicated Diabetes mellitus	15.2 (15–15.4)	12.5 (12.2–12.9)	0.79 (0.77–0.82)	0.00	1.05 (1.02–1.08)	0.00
Complicated Diabetes mellitus	22.4 (22.2–22.7)	40.1 (39.6–40.6)	2.31 (2.26–2.36)	0.00	1.56 (1.52–1.60)	0.00
Uncomplicated hypertension	40.4 (40.1–40.7)	29.8 (29.3–30.2)	0.62 (0.61–0.63)	0.00	1.00 (0.97–1.03)	0.72
Complicated hypertension	22.3 (22–22.6)	44 (43.25–44.6)	2.73 (2.67–2.79)	0.00	1.20 (1.16–1.25)	0.00
Solid cancer	2.5 (2.5–2.6)	3.4 (3.3–3.6)	1.34 (1.28–1.41)	0.00	1.15 (1.08–1.21)	0.00
Dyslipidemia	39.1 (38.7–39.6)	42.7 (42.1–43.3)	1.15 (1.13–1.18)	0.00	0.83 (0.81–0.85)	0.00
Overweight and obesity	24.9 (24.4–25.3)	28.3 (27.7–28.9)	1.19 (1.16–1.22)	0.00	1.14 (1.10–1.17)	0.00
Malnutrition	12.6 (12.3–12.9)	26.3 (25.7–26.9)	2.47 (2.40–2.54)	0.00	2.26 (2.19–2.33)	0.00
Primary immunodeficiency	0.1 (0.1–0.1)	0.1 (0.1–0.2)	1.50 (1.21–1.87)	0.00	1.74 (1.36–2.22)	0.00
Nicotine abuse	6.6 (6.4–6.8)	5.7 (5.5–5.9)	0.86 (0.83–0.89)	0.00	0.82 (0.79–0.86)	0.00
Sickle cell and thalassemia	0.1 (0.1–0.1)	0.09 (0.07–0.1)	0.85 (0.66–1.11)	0.22	0.84 (0.63–1.22)	0.24
Any transplant	0.7 (0.6–0.7)	0.9 (0.8–1)	1.32 (1.21–1.45)	0.00	0.76 (0.68–0.84)	0.00
Substance abuse	2.2 (2.2–2.3)	2.7 (2.6–2.9)	1.20 (1.14–1.27)	0.00	1.00 (0.94–1.07)	0.78
Tuberculosis	0.01 (0.01–0.02)	0.03 (0.02–0.04)	1.77 (1.05–2.99)	0.03	1.29 (0.71–2.35)	0.39
Long term steroid or immunomodulators	12.3 (12–12.5)	16.1 (15.7–16.5)	1.36 (1.32–1.40)	0.00	1.28 (1.24–1.33)	0.00
Asthma	8.2 (8.1–8.4)	6.4 (6.1–6.6)	0.75 (0.73–0.78)	0.00	0.90 (0.87–0.94)	0.00
COPD $	1.6 (1.6–1.7)	2.2 (2–2.3)	1.31 (1.23–1.39)	0.00	1.30 (1.21–1.39)	0.00
Cystic fibrosis	0.02 (0.01–0.03)	0.01 (0.01–0.03)	0.84 (0.46–1.53)	0.57	0.75 (0.37–1.52)	0.43
Other Chronic pulmonary disorders	11.3 (11.1–11.5)	16.3 (15.9–16.6)	1.52 (1.48–1.56)	0.00	1.21 (1.17–1.24)	0.00
Liver disease	3.9 (3.8–4)	10.9 (10.6–11.2)	3.00 (2.91–3.11)	0.00	2.90 (2.83–3.05)	0.00
Rheumatoid arthritis or collagen vascular disease	2.8 (2.8–2.9)	3 (2.8–3.1)	1.04 (0.99–1.10)	0.06	1.05 (0.99–1.10)	0.07
Obstructive sleep apnea	7.3 (7.1–7.5)	10.9 (10.5–11.3)	1.54 (1.49–1.60)	0.00	1.33 (1.28–1.38)	0.00
Elixhauser comorbidity index categories						
≤3	56.6 (56.1–57.1)	23.5 (22.9–24)	Reference			
4–6	35.4 (35.1–35.8)	51.6 (51.1–52.1)	3.51 (3.41–3.60)	0.00		
>6	7.8 (7.6–8)	24.8 (24.2–25.4)	7.60 (7.33–7.88)	0.00		
Mean Elixhauser	3.3 (3.3–3.3)	5.07 (5.03–5.1)	1.43 (1.42–1.44)	0.00		

* Attention deficit hyperactivity disorder, $ Chronic obstructive pulmonary disease, ^ Unweighted ROC for multivariate model = 0.72.

**Table 6:** Logistic Regression for Socioeconomic Predictors of Mortality.

	Survivors 1,385,330 (1,352,424–1,418,236)	Non-Survivors 222,490 (215,758–229,221)	Unadjusted		Adjusted *	
	Proportion (95% CI)	Proportion (95% CI)	OR (95% CI)	*p*-Value	OR (95% CI)	*p*-Value
Age (continuous)	61.7 (61.5–61.9)	72.7 (72.5–72.9)	1.04 (1.04–1.04)	0.00	1.05 (1.04–1.05)	0.00
Age categories						
18–40	14.1 (13.8–14.4)	2 (1.9–2.2)	Reference	0.00		
41–64	37.8 (37.5–38.1)	22.1 (21.6–22.6)	3.99 (3.72–4.28)	0.00		
65 and above	46 (45.6–46.5)	73.7 (73.1–74.3)	10.9 (10.1–11.6)	0.00		
Female sex	49.2 (49–49.4)	41.5 (41–42)	0.73 (0.71–0.74)	0.00	0.69 (0.68–0.71)	0.00
Insurance status						
Private insurance	48 (47.4–48.6)	67.7 (66.9–68.5)	Reference		Reference	
Medicare	15.3 (14.8–15.8)	8.9 (8.4–9.4)	2.48 (2.38–2.58)	0.00	0.97 (0.93–1.02)	0.29
Medicaid	27.9 (27.4–28.4)	15.8 (15.2–16.5)	1.02 (0.97–1.08)	0.33	1.09 (1.04–1.15)	0.00
Self-pay	4 (3.7–4.3)	2.3 (2.1–2.6)	1.03 (0.93–1.14)	0.47	1.15 (1.04–1.27)	0.00
No charge	0.2 (0.2–0.4)	0.1 (0.1–0.2)	0.95 (0.69–1.32)	0.80	0.82 (0.60–1.14)	0.25
Other	4.3 (4–4.5)	4.8 (4.4–5.3)	1.99 (1.82–2.17)	0.00	1.57 (1.44–1.70)	0.00
Race						
Whites	50.4 (49.3–51.5)	53.1 (51.7–54.5)	Reference		Reference	
Blacks	19.3 (18.5–20.2)	17.4 (16.5–18.3)	0.85 (0.82–0.88)	0.00	1.03 (0.99–1.07)	0.12
Hispanics	21.7 (20.7–22.7)	20.2 (19–21.4)	0.88 (0.84–0.92)	0.00	1.26 (1.21–1.31)	0.00
Asian or pacific islander	3.1 (2.9–3.4)	3.4 (3.1–3.8)	1.02 (0.95–1.10)	0.50	1.21 (1.12–1.30)	0.00
Native American	0.9 (0.8–1.1)	1.2 (1–1.5)	1.24 (1.10–1.40)	0.00	1.98 (1.73–2.27)	0.00
Others	4.2 (3.8–4.6)	4.5 (4–5)	1.01 (0.94–1.09)	0.66	1.27 (1.19–1.36)	0.00
Zip Income quartile						
0–25th percentile	33.7 (32.6–34.9)	35.4 (34.1–36.8)	Reference		Reference	
26th to 50th percentile	27.9 (26.4–27.9)	26.7 (25.7–27.6)	0.93 (0.90–0.97)	0.00	0.89 (0.85–0.92)	0.00
51st to 75th percentile	22.4 (21.7–23.1)	21.2 (20.3–22.1)	0.90 (0.86–0.94)	0.00	0.82 (0.78–0.85)	0.00
76th to 100th percentile	16.6 (15.7–17.5)	16.5 (15.4–17.6)	0.94 (0.90–0.99)	0.03	0.78 (0.74–0.82)	0.00
Hospital bed size						
Small	24 (23–25.1)	22.2 (20.9–23.5)	Reference		Reference	
Medium	28.7 (27.8–29.8)	30.5 (29.2–31.9)	1.14 (1.08–1.21)	0.00	1.13 (1.07–1.20)	0.00
Large	47.1 (45.9–48.3)	47.2 (45.7–48.7)	1.08 (1.03–1.14)	0.00	1.14 (1.08–1.20)	0.00
Hospital type						
Rural	9.5 (9–10)	7.8 (7.2–8.4)	Reference		Reference	
Urban non-teaching	18.7 (17.9–19.5)	17.7 (16.7–18.7)	1.15 (1.07–1.22)	0.00	1.25 (1.16–1.34)	0.00
Urban teaching	71.7 (70.8–72.6)	74.4 (73.2–75.5)	1.26 (1.19–1.33)	0.00	1.38 (1.29–1.47)	0.00
Hospital region						
Northeast	17.6 (16.6–18.6)	23 (21.5–24.5)	Reference		Reference	
Midwest	22.5 (21.5–23.4)	20.2 (19.1–21.3)	0.68 (0.64–0.73)	0.00	0.65 (0.60–0.69)	0.00
South	41.5 (40.4–42.7)	38.3 (36.9–39.8)	0.70 (0.66–0.75)	0.00	0.70 (0.66–0.75)	0.00
West	18.2 (17.3–19.2)	18.3 (17.2–19.5)	0.76 (0.71–0.82)	0.00	0.76 (0.71–0.82)	0.00
Hospital control						
Government, non-federal	12 (11.3–12.7)	11.9 (11–12.9)	Reference		Reference	
Private, not-profit	73.3 (72.4–74.3)	72.9 (71.6–74.2)	1.00 (0.93–1.06)	0.94	0.88 (0.83–0.94)	0.00
Private, invest-own	14.5 (13.8–15.3)	15 (14–16.1)	1.04 (0.96–1.12)	0.28	0.92 (0.85–0.99)	0.04

* Unweighted ROC for multivariate model = 0.74.

**Table 7:** Logistic Regression for Comorbid Medical Predictors of Mortality.

	Survivors	Non-Survivors	Unadjusted		Adjusted ^&^	
	Proportion (95% CI)	Proportion (95% CI)	OR (95% CI)	*p*-Value	OR (95% CI)	*p*-Value
Stroke	1 (1–1)	2.9 (2.7–3.1)	2.84 (2.65–3.04)	0.00	2.42 (2.22–2.65)	0.00
CKD	18.7 (18.5–19)	32.7 (32.1–33.3)	2.10 (2.05–2.15)	0.00	1.19 (1.14–1.24)	0.00
Severe IQ disability, iodine def, downs syndrome and autism	0.1 (0.1–0.1)	0.1 (0.09–0.1)	0.86 (0.65–1.15)	0.31	1.31 (0.97–1.78)	0.07
ADHD	0.3 (0.3–0.3)	0.07 (0.05–0.1)	0.20 (0.14–0.29)	0.00	0.68 (0.48–0.97)	0.03
Cerebral palsy	0.3 (0.3–0.3)	0.2 (0.2–0.3)	0.84 (0.70–1.00)	0.00	1.48 (1.21–1.81)	0.00
Paralysis	0.6 (0.5–0.6)	1.1 (1–1.2)	1.85 (1.67–2.05)	0.00	0.95 (0.83–1.09)	0.53
Heart failure	14.6 (14.4–14.8)	27.2 (26.7–27.8)	2.18 (2.12–2.24)	0.00	1.25 (1.20–1.30)	0.00
Cardiomyopathy	2.6 (2.5–2.7)	4.2 (4–4.5)	1.65 (1.56–1.73)	0.00	1.12 (1.05–1.18)	0.00
Ischemic heart disease	16.8 (16.5–17.1)	25 (24.4–25.5)	1.64 (1.60–1.68)	0.00	0.97 (0.94–1.00)	0.09
Mood or psychotic disorders	0.8 (0.8–0.9)	0.5 (0.4–0.6)	0.63 (0.55–0.72)	0.00	0.68 (0.59–0.80)	0.00
HIV/AIDS	0.6 (0.5–0.6)	0.5 (0.4–0.6)	0.86 (0.73–1.01)	0.10	0.93 (0.78–1.11)	0.43
Uncomplicated Diabetes mellitus	14.8 (14.6–15)	13.8 (13.4–14.2)	0.92 (0.89–0.95)	0.00	1.11 (1.07–1.15)	0.00
Complicated Diabetes mellitus	24.9 (24.7–25.2)	35 (34.4–35.6)	1.62 (1.58–1.66)	0.00	1.32 (1.29–1.36)	0.00
Uncomplicated Hypertension	39 (38.7–39.3)	31.9 (31.3–32.5)	0.73 (0.71–0.75)	0.00	0.79 (0.76–0.82)	0.00
Complicated Hypertension	24.7 (24.4–25)	41.8 (41.2–42.5)	2.19 (2.13–2.24)	0.00	0.94 (0.90–0.98)	0.01
Solid cancer	2.5 (2.4–2.6)	4.1 (3.9–4.3)	1.63 (1.54–1.72)	0.00	1.22 (1.15–1.30)	0.00
Dyslipidemia	39.4 (39–39.8)	43.1 (42.4–43.8)	1.16 (1.13–1.19)	0.00	0.78 (0.76–0.80)	0.00
Overweight and obesity	26.3 (25.8–26.7)	21.7 (21.1–22.3)	0.77 (0.75–0.80)	0.00	1.07 (1.03–1.11)	0.00
Malnutrition	14 (13.7–14.3)	25.4 (24.7–26)	2.08 (2.03–2.14)	0.00	1.67 (1.62–1.73)	0.00
Primary immunodeficiency	0.1 (0.1–0.1)	0.1 (0.1–0.2)	1.32 (1.04–1.67)	0.03	1.61 (1.25–2.06)	0.00
Nicotine abuse	6.8 (6.6–7)	3.9 (3.7–4.1)	0.56 (0.53–0.59)	0.00	0.68 (0.64–0.72)	0.00
Sickle cell and thalassemia	0.1 (0.1–0.1)	0.05 (0.03–0.08)	0.44 (0.29–0.67)	0.00	0.52 (0.34–0.81)	0.00
Any transplant	0.7 (0.7–0.8)	0.8 (0.7–0.9)	1.06 (0.94–1.19)	0.30	0.95 (0.83–1.08)	0.44
Substance abuse	2.4 (2.3–2.5)	1.8 (1.7–2)	0.75 (0.69–0.80)	0.00	0.81 (0.74–0.88)	0.00
Tuberculosis	0.01 (0.01–0.02)	0.02 (0.01–0.05)	1.52 (0.84–2.77)	0.16	1.45 (0.71–2.92)	0.29
Long term steroid or immunomodulators	13.3 (13–13.5)	12.2 (11.8–12.6)	0.91 (0.88–0.94)	0.00	1.37 (1.32–1.43)	0.00
Asthma	8.3 (8.2–8.5)	4.6 (4.4–4.8)	0.53 (0.50–0.55)	0.00	0.81 (0.77–0.85)	0.00
COPD	1.6 (1.6–1.7)	2.5 (2.3–2.7)	1.52 (1.42–1.62)	0.00	1.27 (1.18–1.37)	0.00
Cystic fibrosis	0.02 (0.01–0.02)	0.02 (0.01–0.04)	0.98 (0.51–1.89)	0.97	1.87 (0.92–3.81)	0.08
Other Chronic pulmonary disorders	11.5 (11.3–11.7)	18.1 (17.7–18.6)	1.70 (1.65–1.75)	0.00	1.20 (1.17–1.24)	0.00
Liver disease	4.7 (4.6–4.8)	9.8 (9.5–10.2)	2.19 (2.11–2.27)	0.00	2.61 (2.50–2.72)	0.00
Rheumatoid arthritis or collagen vascular disease	2.8 (2.8–2.9)	3.1 (3–3.3)	1.10 (1.04–1.17)	0.00	1.10 (1.03–1.18)	0.00
Obstructive sleep apnea	8.2 (8–8.4)	7.5 (7.2–7.8)	0.91 (0.87–0.94)	0.00	0.89 (0.85–0.93)	0.00
Elixhauser comorbidity index categories						
≤3	53.3 (52.9–53.8)	24.4 (23.8–25.1)	Reference			
4–6	37 (36.7–37.3)	51.2 (50.6–51.8)	3.01 (2.91–3.11)	0.00		
>6	9.5 (9.3–9.7)	24.2 (23.6–24.9)	5.52 (5.29–5.76)	0.00		
Elixhauser comorbidity index (numerical scale)	3.5 (3.4–3.5)	5 (4.9–5)	1.34 (1.33–1.35)	0.00		
Severe sepsis	4.1 (3.9–4.3)	8.1 (7.7–8.6)	2.06 (1.96–2.17)	0.00		
Septic shock	3 (2.9–3.1)	33.7 (32.9–34.5)	16.4 (15.8–17)	0.00		
Acute kidney injury	23.2 (22.9–23.5)	62.1 (61.5–62.8)	5.40 (5.25–5.56)	0.00		
Urinary filtration	3.6 (3.5–3.7)	14.5 (14–15)	4.52 (4.33–4.71)	0.00		
RBC transfusion	2.7 (2.5–2.8)	9.1 (8.6–9.6)	3.58 (3.41–3.75)	0.00		
Vasopressor need	1 (0.9–1.1)	12.2 (11.3–13.2)	13.1 (12.2–14)	0.00		
Acute respiratory failure	50.4 (49.9–51)	71.2 (70.2–72)	2.42 (2.31–2.53)	0.00		
BIPAP/CPAP and HFNC ^	4.1 (3.9–4.3)	18.4 (17.6–19.4)	5.21 (4.99–5.45)	0.00		
Mechanical Ventilation	4.1 (4–4.3)	47.8 (47–48.7)	21 (20.1–21.9)	0.00		
Acute liver failure	0.6 (0.5–0.6)	6.2 (6–6.5)	10.8 (10.2–11.6)	0.00		
Extracorporeal membrane oxygenation	0.1 (0.08–0.1)	0.7 (0.6–0.9)	7.38 (6.17–8.84)	0.00		
Cardiac arrest	0.3 (0.3–0.3)	17.2 (16.6–17.8)	57.9 (53.4–62.7)	0.00		
Composite complications *	14 (13.7–14.3)	71.3 (70.5–72)	15.1 (14.6–15.7)	0.00		

^&^ Unweighted ROC for multivariate model = 0.74, * urinary filtration, Acute Liver Failure, RBC Transfusion, BIPAP/CPAP or HFNC, Mechanical Ventilation, Vasopressor, Septic Shock, ECMO and Cardiac Arrest, ^ BIPAP: Bilevel Positive Airway Pressure, CPAP: Continuous Positive Airway Pressure, HFNC: High Flow Nasal Cannula.

## Data Availability

NIS dataset is available at https://www.hcup-us.ahrq.gov/nisoverview.jsp (accessed on 25 November 2022).

## Supplementary Materials

The following supporting information can be downloaded at: https://www.mdpi.com/article/10.3390/medsci10040067/s1, Table S1: ICD-10 codes of comorbidities; Table S2: ICD-10 codes of complications.
