# Supplementary material for: Population Risk Factors for Severe Disease and Mortality in COVID-19 in the United States during the Pre-Vaccine Era: A Retrospective Cohort Study of National Inpatient Sample
Source: Med Sci (Basel). 2022 Dec 4;10(4):67. doi: 10.3390/medsci10040067 (PMC9788467; doi:10.3390/medsci10040067)
Supplement: Supplementary file 1 [file medsci-10-00067-s001.zip › medsci-2033916-supplementary.pdf]

Supplementary files

ICD-10 codes.

Table S1: ICD-10 codes of comorbidities

COVID-19	U07.1
COVID-19 pneumonia	U071 & J12.89
COVID-19 bronchitis and LRTI	U07.1 & J20.8 or J40 or J22 or J98.8
COVID-19 ARDS	U07.1 & J80
Asymptomatic COVID-19	U07.1 while excluding the above categories
Asthma	J45
COPD	J43, J44, J41, J42
Chronic pulmonary disorders	I27.8, I27.9, J40.x – J47.x, J60.x – J67.x, J68.4, J70.1, J70.3
Cystic fibrosis	E84
Other chronic pulmonary disorders	Chronic pulmonary disorders after excluding asthma, COPD and cystic fibrosis
Stroke	I67.81, I67.82, I63, G46, I69
CKD	N18
Severe IQ disability, iodine deficiency, downs syndrome and autism	F72, F73, E00, Q90, F84
ADHD	F90
Cerebral palsy	G80
Paralysis	G81, G82, G83
Heart failure	I50
Cardiomyopathy	I42,43
Ischemic heart disease	I20, I21, I22, I23, I24, I25
Mood or psychotic disorders	F28 to F33, F39, F21 to F25
HIV/AIDS	B20, Z21
Uncomplicated DM	E10.0, E10.1, E10.9, E11.0, E11.1, E11.9, E12.0, E12.1, E12.9, E13.0, E13.1, E13.9, E14.0, E14.1, E14.9
Complicated DM	E10.2 - E10.8, E11.2 - E11.8, E12.2 - E12.8, E13.2 - E13.8, E14.2 - E14.8
Uncomplicated hypertension	I10.x
Complicated hypertension	I11.x - I13.x, I15.x
Solid cancer	C00.x - C26.x, C30.x - C34.x, C37.x - C41.x, C43.x, C45.x - C58.x, C60.x - C76.x, C97.x C77.x - C80.x
Dyslipidemia	E78.00, E78.01, E78.1, E78.2, E78.3, E78.41, E78.49, E78.5
Overweight and obesity	E66
Malnutrition	E43, E44, E46, D64
Primary immunodeficiency	D80 to D84
Nicotine abuse	F17, Z57.31, Z72.0, Z77.22, T65.2
Sickle cell and thalassemia	D56, D57
Any transplant	Z94, T86
Substance abuse	F11 to F16, F18, F19, R78
Tuberculosis	A15, A17 to A19
Long term steroid or immunomodulators	Z92.241, Z92.25, Z79.52

Liver disease	B18.x, I85.x, I86.4, I98.2, K70.x, K71.1, K71.3 - K71.5, K71.7, K72.x - K74.x, K76.0, K76.2 - K76.9, Z94.4
Rheumatoid arthritis or collagen vascular disease	L94.0, L94.1, L94.3, M05.x, M06.x, M08.x, M12.0, M12.3, M30.x, M31.0 - M31.3, M32.x - M35.x, M45.x, M46.1, M46.8, M46.9
Obstructive sleep apnea	G47.33

Table S2: ICD-10 codes of complications

	ICD-10 codes
Severe sepsis	R65.20
Septic shock	R65.21
Acute kidney injury	N17
Urinary filtration	5A1D
RBC transfusion	30233N, 30243N, 30243P, 30233P
Vasopressor need	3E030XZ, 3E033XZ, 3E040XZ, 3E043XZ, 3E053XZ, 3E060XZ, 3E063XZ, 3E050XZ
Acute respiratory failure	J96
BIPAP/CPAP and HFNC^	5A09357, 5A09358, 5A0935A, 5A09457, 5A09458, 5A0945A, 5A09557, 5A09558, 5A0955A
Mechanical Ventilation	0BH17EZ, 0BH18EZ, 0BH13EZ
Acute liver failure	K72.9, K72.0
Extracorporeal membrane oxygenation	5A15
Cardiac arrest	I46
Composite complications	Urinary filtration, acute liver failure, RBC transfusion, HFNC and BIPAP/CPAP, Mechanical ventilation, vasopressor use, septic shock.
